# Supplementary figures and images for: Itraconazole resistance in Madurella fahalii linked to a distinct homolog of the gene encoding cytochrome P450 14-α sterol demethylase (CYP51)
Source: PLoS Negl Trop Dis. 2025 Mar 27;19(3):e0012623. doi: 10.1371/journal.pntd.0012623 (PMC11964275; doi:10.1371/journal.pntd.0012623)

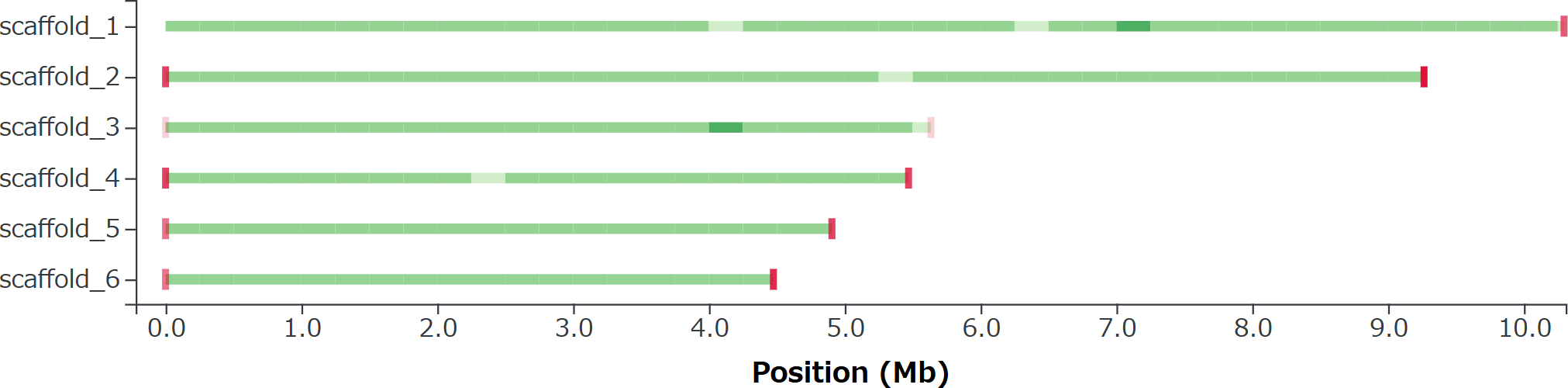

Supplement: S1 Fig — The figure was generated using Tapestry (https://github.com/johnomics/tapestry) by mapping long reads, which were used as input for Flye assembly. Regions corresponding to telomeric sequences (CCCTAA/TTAGGG) are indicated in red and the opacity represents the number of telomeric repeats. (TIF) [file pntd.0012623.s003.tif]

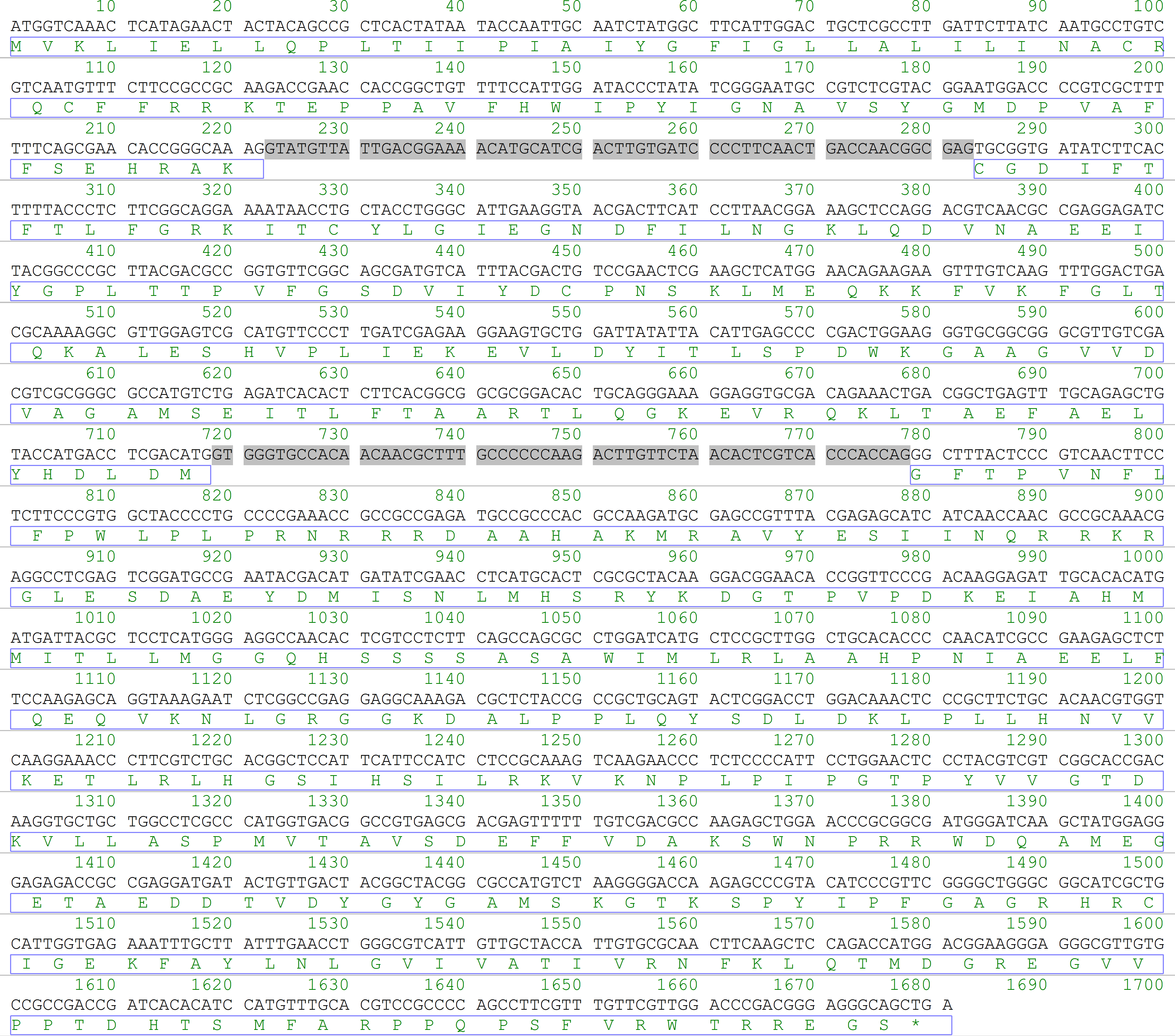

Supplement: S2 Fig — Introns were highlighted with gray, and encoded amino acids were represented under the corresponding nucleotide sequences. (TIF) [file pntd.0012623.s004.tif]

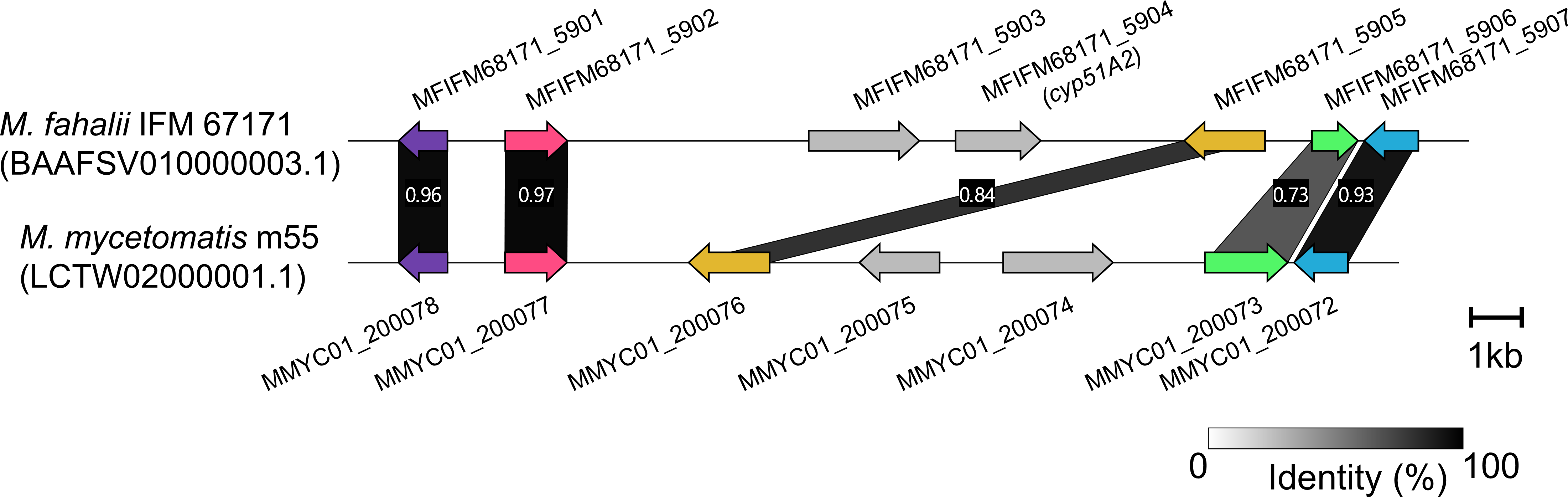

Supplement: S3 Fig — The amino acids alignment and the generation of the image was performed by Clinker (https://github.com/gamcil/clinker). The homologous protein-coding gene models were drawn as the arrows in the same color and their similarity in amino acids were labeled between them. (TIF) [file pntd.0012623.s005.tif]

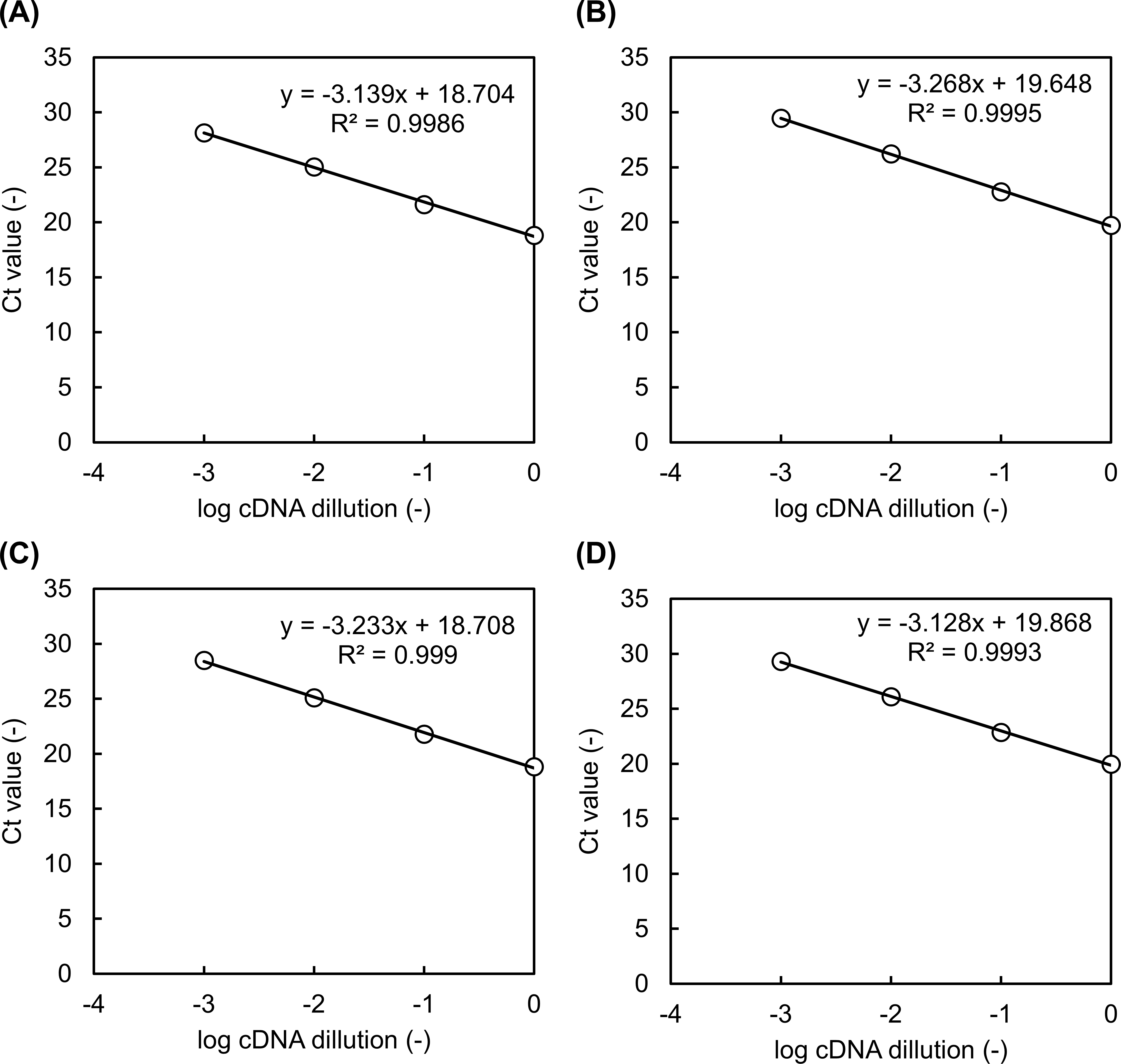

Supplement: S4 Fig — cDNA solutions were serially diluted 10-fold and were used as input. The x-axis represents the log10 of the DNA dilution factor, while the y-axis represents the Ct values. (TIF) [file pntd.0012623.s006.tif]

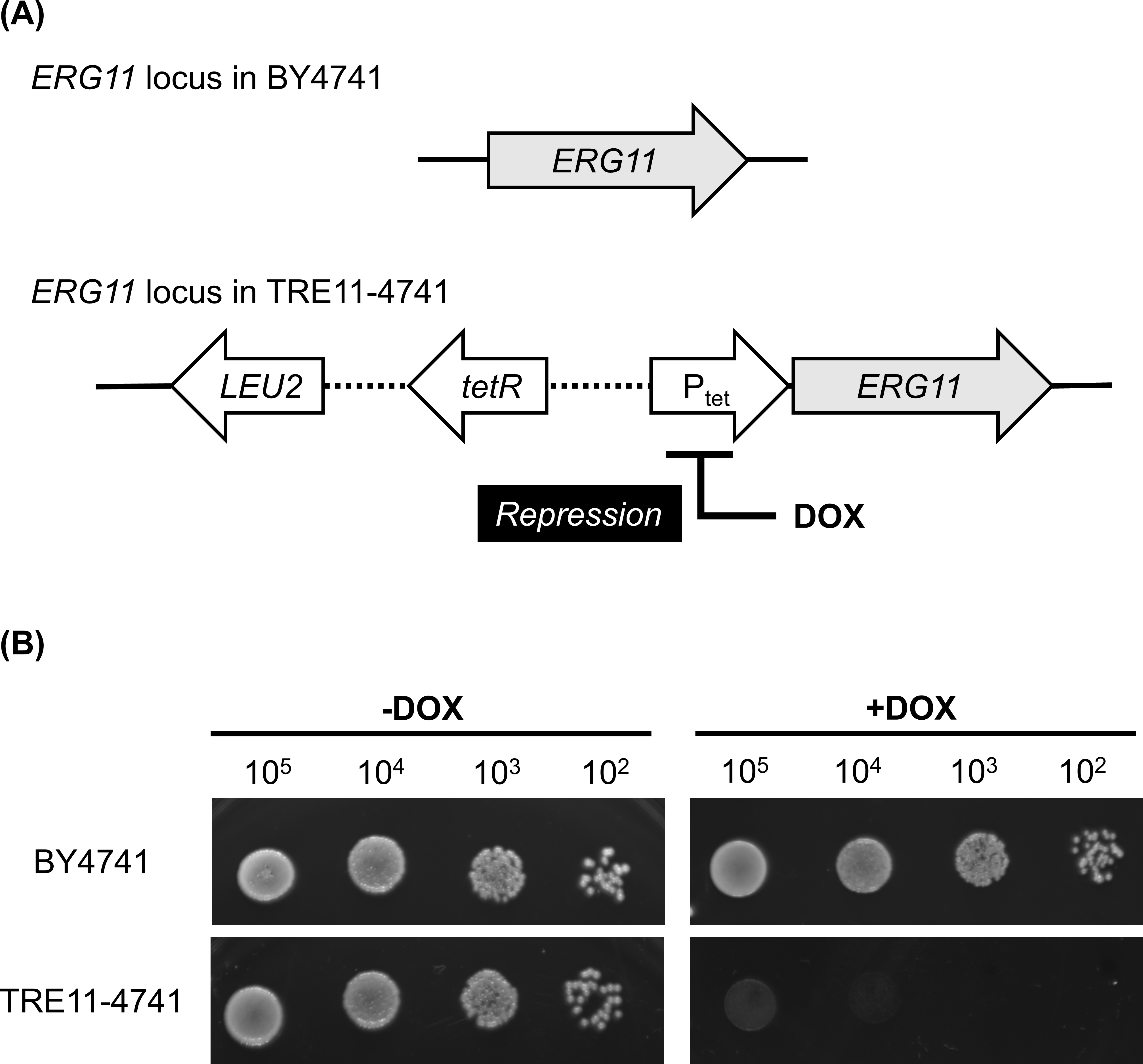

Supplement: S5 Fig — (A) Schematic representation of ERG11 locus in strains BY4741 and TRE11-4741. In the genome of TRE11-4741, LEU2 gene, PCMV -tTA-TADH1 cassette (tetR) and tetO7 -PCYC1 -UAS cassette (Ptet) were integrated into the upstream region of ERG11 ORF in comparison with that of strain BY4741.The genomic region and vector backbone were represented as solid and dotted lines, respectively. The expression of ERG11 by strain TRE11-4741 is repressed by the addition of DOX. (B) Restricted growth of strain TRE11-4741 by the addition of DOX. Yeast cells were serially diluted from 105 to 102 by 10-fold and cultivated on YPD media without DOX (-DOX) or with 10 mg/L DOX (+DOX) for 2 days. (TIF) [file pntd.0012623.s007.tif]

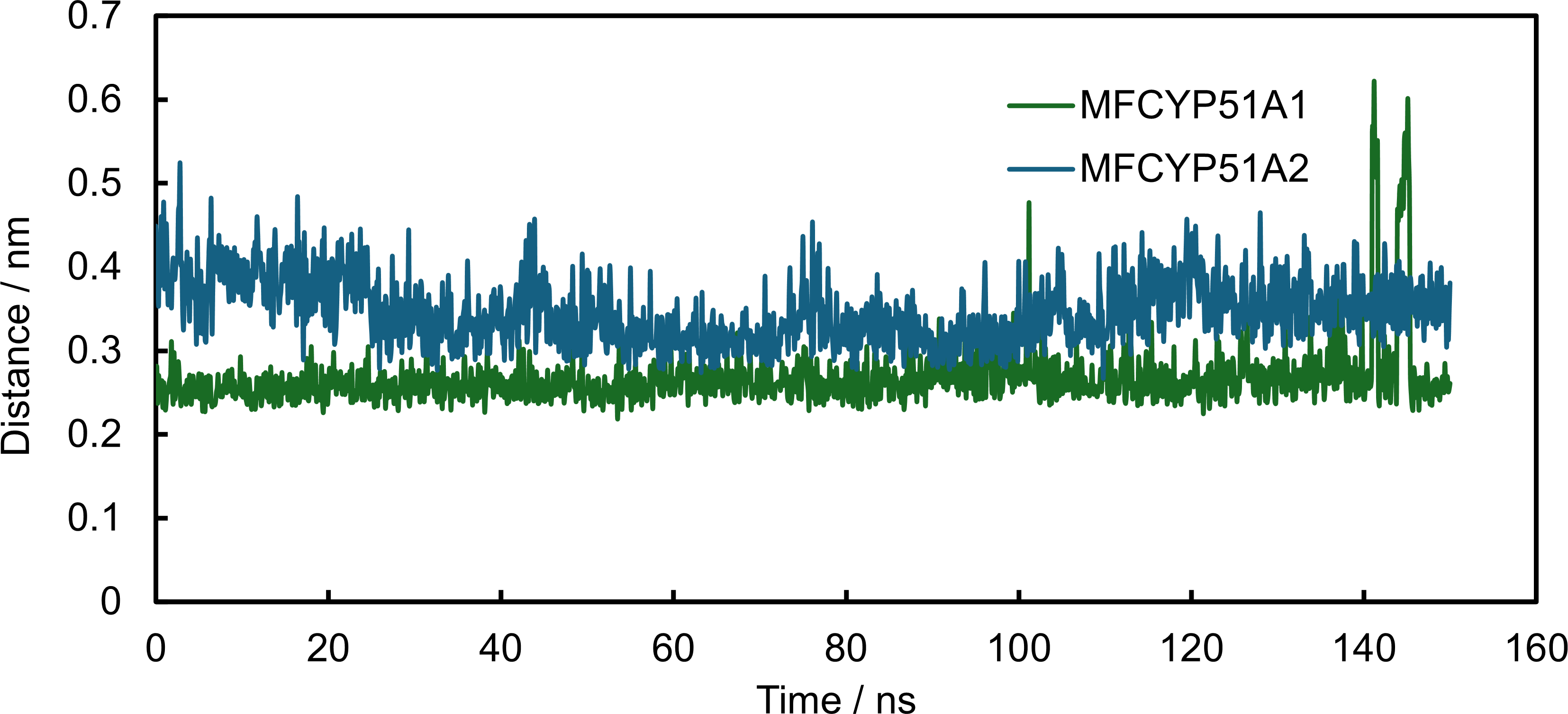

Supplement: S6 Fig — Green and blue plots represent the data for MFCYP51A1 and MFCYP51A2, respectively. (TIF) [file pntd.0012623.s008.tif]
