# Supplementary material for: Itraconazole resistance in Madurella fahalii linked to a distinct homolog of the gene encoding cytochrome P450 14-α sterol demethylase (CYP51)
Source: PLoS Negl Trop Dis. 2025 Mar 27;19(3):e0012623. doi: 10.1371/journal.pntd.0012623 (PMC11964275; doi:10.1371/journal.pntd.0012623)
Supplement: Attachment — Submitted filename: Response_letter-2.docx [file pntd.0012623.s010.docx]

**Editor**

We thank you for your checking our manuscript. We have revised it according to your comments.

**4)** We have noticed that you have uploaded Supporting Information files, but you have not included a list of legends. Please add a full list of legends for your Supporting Information files after the references list.

**(A4)**

We added a full list of legends for the Supporting Information files after the references list.

**5)** We have noticed that you have cited "Table S3" in the manuscript file. However, there are no corresponding file uploaded to the submission. Please ensure that all files are present to ensure that your paper is fully reviewed.

**(A5)**

We changed "Table S3" to "Table S2".

**7)** Please amend your detailed Financial Disclosure statement. This is published with the article, therefore should be completed in full sentences and contain the exact wording you wish to be published.

**(A7)**

We stated “The funders had no role in study design, data collection and analysis, decision to publish, or preparation of the manuscript.”

We confirmed 1) - 3), 6) and 8).

**Reviewer1**

We thank you for your invaluable comments and suggestions. We have revised the manuscript according to your comments. Hereafter, the revised manuscript is referred to as “2nd manuscript.” We highlighted the changes based on the reviewers’ comments using red letters, for your review.

**(Q1)**

One concern related to the gene duplication phenomenum is whehther this is a strain-specific observation or it is a genetic feature bradly found in this species. The authors are encouraged to assess the presence of these two copies in other isolates of this species.

**(A1)**

We thank for your valuable suggestions. Following your comments, we performed PCR analysis to detect *Mfcyp51A2* in other *Madurella fahalii* strains, and added its results to 2nd manuscript as Fig. 2. In addition, the description of the experiments was added as follows:

**2nd Manuscript: Page 5, Lines 111-115**

*M. mycetomatis* IFM 46458, *M. fahalii* IFM 68171 (preserved as MRC No.13 at the Mycetoma Research Center, Khartoum, Sudan), IFM 68170 (MRC No.9) and IFM 68242 (MRC No. 25) were grown and maintained on Sabouraud medium (FUJIFILM Wako Pure Chemical Corporation, Osaka, Japan) containing 1.5% (w/v) agar.

**2nd Manuscript: Page 10, Lines 218-220**

A primer pair, Pr1-Pr2, was used for PCR amplification to detect the entire ORF from genomic DNA and the corresponding cDNA regions.

**2nd Manuscript: Page 15, Lines 332-335**

Furthermore, *Mfcyp51A2* was successfully amplified from the genomic DNA of other *M. fahalii* strains (IFM 68170 and IFM 68242), while it was not detected in *M. mycetomatis* IFM 46458, as shown in Fig. 2.

Fig. 2

**2nd Manuscript: Pages 21-22, Lines 450-455**

Moreover, we have confirmed that *Mfcyp51A2* is conserved among *M. fahalii* strains (Fig. 2), suggesting that *Mfcyp51A2* as well as *Mfcyp51A1* could be important targets for discussing azole resistance in this species, such as the variability in azole resistance in clinical isolates and molecular genetic analysis of the existing resistant strains.

**(Q2)**

In addition, no effort was shown to assambly contigns and establish loci localization, which is esential to analysis gene syntheny.

**(A2)**

According to your comment, we newly added the data and descriptions to evaluate the quality of genome assembly (Supplementary Figs. S1 and S3), as follows:

**2nd Manuscript: Page 14, Lines 311-317**

Five of the scaffolds contained telomeric sequences at both ends, while the remaining scaffold a telomeric sequence at one end, as shown in Fig. S1. Genome annotation identified 11,334 protein-coding genes, 180 tRNAs, and 79 rRNAs. The BUSCO v.5.5.0 [45] score based on the eukaryote and sordariomycetes databases were 100% and 99.6%, respectively. These results indicated that a high-quality genome assembly of *M. fahalii* IFM 68171 was obtained

**2nd Manuscript: Page 15, Lines 330-332**

Notably, no homologous *Mfcyp51A2* gene exists in the genome of *M. mycetomatis*, although its adjacent gene locus is conserved, as depicted in Fig. S3.

**(Q3)**

Related with the expression assays, there is no evidence nor reference supporting the use of the ACT1 gene to normalize gene expression assays. Actin is a highly dinamic gene and may not of stable, and constant expression in this species.

In addition, amplification efficiency of primer pairs used in RT-qPCR should be inclued in as part for the manuscript.

**(A3)**

We newly designed qPCR primer targeting tubulin gene as a reference and preformed qPCR assay. Then, *cyp51A* genes are quantified using two reference genes (actin and tubulin), as shown in Table 2. In addition, the primers’ efficiency was determined by plotting the standard curves. The results and descriptions are added as follows:

**2nd Manuscript: Page 10, Lines 220-222**

The transcription levels of *cyp51A* were measured using primer pairs Pr3-Pr4 or Pr5-Pr6, and they were normalized against actin and tubulin, amplified with primer pairs Pr7-Pr8 and Pr9-Pr10, respectively.

**2nd Manuscript: Page 17, Lines 358-367**

The transcriptional activity of the *Mfcyp51A* genes in *M. fahalii* was analyzed both in the presence and absence of 2 mg/L itraconazole. To estimate the transcriptional levels, two housekeeping genes (actin and tubulin) [13] were used for internal standards. The primer efficiency was determined using cDNA solutions by plotting a standard curve that were serially diluted by 10-fold, as shown in Fig. S4 and Table S3. The results of qPCR was summarized in Table 2. In the absence of itraconazole, the transcriptional level of *Mfcyp51A1* was 1.89 times higher than that of *Mfcyp51A2*. However, upon the addition of itraconazole, transcription levels of *Mfcyp51A1* increased by 1.25- or 1.96-fold while those of *Mfcyp51A2* increased by 2.00- or 3.14-fold.

**(Q4)**

It is strange that sensitivity assays were not performed following conventional assays in RPMI broth. It is known that growing fungal cells in rich media affect the susceptibility profiles. For both, the complementation assays and the expression analysis, it should be relevant to present the result generated with the other azoles tested. Moreover, a control with a non-azole antifungal drug should also be included in the analysis. The most relevant issue, the evidence is not solid enough to support the role of this gene copy in itraconazole resistance. Actually, results suggest a modest upregulation in presence of the azole, and the complementation assays showed partial results. A stronger data set is required to link this gene with the itraconazole resistance in Madurella fahalii.

**(A4)**

In this study, we focus on- and discuss the potential contribution of *Mfcyp51A2* to ITCZ resistance using heterologous expression. Thus, the azole susceptibility was qualitatively assessed. In particular, the important points of this article is that transcription of *cyp51A2* in azole-resistant *M. fahalii* isolate was confirmed and that the heterologous expression in yeast doubtlessly affect the ITCZ resistance. In addition, as you mentioned, the direct analysis of these function in *Madurella* should be perfomed, but we described it in discussion section as future perspectives. Taken together, our manuscript fulfills the objectives “to discuss about the potential contribution of Mfcyp51A2 to ITCZ resistance”

**(Q5)**

Moreover, we added the results of Molecular Dynamics Simulations to explain differences of the ITCZ resistance in CYP proteins. We are convinced that these data will give “a stronger data set”.

**(A5)**

Thank you for your absolutely valuable comments to improve our manuscripts. In accordance with your suggestion, we performed MD Simulations and added the results as follows:

**2nd Manuscript: Pages 12-13, Lines 276-300**

The 3D models of MFCYP51A1 and MFCYP51A2 were generated using AlphaFold2 v.2.3.2 with a default parameter [38]. These models were refined using openMM v.8.2.0 to minimize the local energy [39]. The 3D structure of itraconazole was retrieved from PubChem (ID: 55283), and used for docking simulation by AutoDock Vina v.1.1.2 [40]. The optimal structure of protein-ligand complex was chosen based on the crystal structure of 14-alpha sterol demethylase derived from *A. fumigatus* Af293, which was retrieved from PDB (ID: 6CR2).

Molecular dynamics (MD) simulations were conducted using AmberTools v.23.4 [41] and GROMACS v.2024.4 [42]. For the force fields, FF19SB was applied to proteins, TIP3P to water molecules, and GAFF2 to itraconazole. Regarding heme, a cofactor located in the active center of CYP51, quantum mechanical calculations were performed by Gaussian v.16 (Gaussian, Inc., Wallingford CT, USA) using the density functional theory (DFT) at the B3LYP/6-31G* level [43], and the resultant model were subsequently integrated into the MD simulations. The system was equilibrated under in the NVT ensemble for 1 ns, followed by NPT equilibration to stabilize the pressure at 1 bar and temperature at 300 K. Finally, the MD simulations were run for 150 ns, during which the temperature was maintained at 300 K using the V-rescale thermostat, and the pressure at 1 bar using the Parrinello−Rahman barostat. Particle Mesh Ewald (PME) was used to calculate electrostatic interactions, with cutoff distances of 10 Å for Coulombic, electrostatic, and van der Waals interactions. For the visualization and analysis of data, PyMOL v.3.0.0 (http://www.pymol.org/pymol) was used. The total binding energy between CYP51 and itraconazole were calculated using gmx_MMPBSA v.1.6.2 [44].

**2nd Manuscript: Pages 19-20, Lines 396-406**

The protein models of MFCYP51A1 and MFCYP51A2 were successfully constructed using AlphaFold2 and refined with OpenMM. The results indicated that the active sites of both enzymes are well-conserved, as shown in Fig. 6. Docking simulations demonstrated that itraconazole binds to the active sites of both MFCYP51A1 and MFCYP51A2, as shown in Fig. 7. However, the orientation of the nitrogen atom in itraconazole relative to the heme iron differs between the two proteins, as shown in Fig. 7. Furthermore, MD simulations demonstrated that the average distance between the heme iron and the nitrogen atom in itraconazole was 2.67 Å for MFCYP51A1 and 3.47 Å for MFCYP51A2, as shown in Fig. S6. The total binding energies for itraconazole of MFCYP51A1 and MFCYP51A2 were -71.38 and -71.69 kcal/mol, respectively.

**2nd Manuscript: Pages 19-20, Lines 432-440**

Regarding protein modeling, the result of MD simulation demonstrated that the distance between heme iron and the nitrogen atom of itraconazole in MFCYP51A2 was greater than MFCYP51A1, while the total binding energies of the two enzymes are nearly equal (Fig. 7). Previous studies reported that the iron-nitrogen distance is an important factor to form the Fe-N coordination bond essential for azole inhibition [51,52]. Thus, our result suggests that MFCYP51A2 exhibits a weak interaction with itraconazole in comparison with MFCYP51A1, which is consistent with the differences in itraconazole susceptibility between *S. cerevisiae* transformants expressing *cyp51A* genes.

**Other Reactions**

According to the other reviewer’s comments, we revised the manuscript as described as follows:

**(R1)**

A new author (Dr. Wei CAO) was added into the authorship due to his total contribution to protein modelling and calculations.

**(R2)**

The Table 1 in previous version manuscript, which lists the primer sequences, was transferred into Supplementary Table (Table S1).

**Reviewer2**

We thank you for your invaluable comments and suggestions. We have revised the manuscripts according to your comments. Hereafter, the revised manuscript is referred to as “2nd manuscript.” We highlighted the changes based on the reviewers’ comments using red letters, for your review.

**(Q1)**

While this is a well written manuscript describing a novel finding of potential clinical significance, my major concern is that the Mfcyp51A2 gene was only identified in a single strain (maybe two?) of *M. fahalii*.

It is possible that this is a rather anomalous finding and the results presented in this manuscript are not extrapolatable to other *M. fahalii* strains or clinical variants. Given that the authors know the sequence for the Mfcyp51A2 gene, it should be a simple matter to design specific primers for PCR amplification to test other strains to determine if this gene is common to clinical isolates of *M. fahalii*. This additional info would greatly enhance the impact of the manuscript.

**(A1)**

We thank for your valuable suggestions. Following your comments, we performed PCR analysis to detect *Mfcyp51A2* in other *Madurella fahalii* strains, and added its results to 2nd manuscript as Fig. 2. In addition, the description of the experiments was added as follows:

**2nd Manuscript: Page 5, Lines 111-115**

*M. mycetomatis* IFM 46458, *M. fahalii* IFM 68171 (preserved as MRC No.13 at the Mycetoma Research Center, Khartoum, Sudan), IFM 68170 (MRC No.9) and IFM 68242 (MRC No. 25) were grown and maintained on Sabouraud medium (FUJIFILM Wako Pure Chemical Corporation, Osaka, Japan) containing 1.5% (w/v) agar.

**2nd Manuscript: Page 10, Lines 218-220**

A primer pair, Pr1-Pr2, was used for PCR amplification to detect the entire ORF from genomic DNA and the corresponding cDNA regions.

**2nd Manuscript: Page 15, Lines 332-335**

Furthermore, *Mfcyp51A2* was successfully amplified from the genomic DNA of other *M. fahalii* strains (IFM 68170 and IFM 68242), while it was not detected in *M. mycetomatis* IFM 46458, as shown in Fig. 2.

**2nd Manuscript: Pages 21-22, Lines 450-455**

Moreover, we have confirmed that *Mfcyp51A2* is conserved among *M. fahalii* strains (Fig. 2), suggesting that *Mfcyp51A2* as well as *Mfcyp51A1* could be important targets for discussing azole resistance in this species, such as the variability in azole resistance in clinical isolates and molecular genetic analysis of the existing resistant strains.

**(Q2)**

Given the reduced sequence homology between Mfcyp51A1 and Mfcyp51A2 (~70%), the question remains if the enzyme is more inherently resistance to azole inhibition due to differences in the ligand binding site, or if it is simply due to increased expression of the second enzyme. The authors briefly touched on this issue in the Discussion, but it would improve the manuscript for them to comment on this aspect of the study further in their discussion and provide any additional data to support their supposition, should they indeed have it.

**(A2)**

Thank you for your absolutely valuable comments to improve our manuscripts. In accordance with your suggestion, we performed MD Simulations and discussed the differences in their susceptibility as follows:

**2nd Manuscript: Pages 12-13, Lines 276-300**

The 3D models of MFCYP51A1 and MFCYP51A2 were generated using AlphaFold2 v.2.3.2 with a default parameter [38]. These models were refined using openMM v.8.2.0 to minimize the local energy [39]. The 3D structure of itraconazole was retrieved from PubChem (ID: 55283), and used for docking simulation by AutoDock Vina v.1.1.2 [40]. The optimal structure of protein-ligand complex was chosen based on the crystal structure of 14-alpha sterol demethylase derived from *A. fumigatus* Af293, which was retrieved from PDB (ID: 6CR2).

Molecular dynamics (MD) simulations were conducted using AmberTools v.23.4 [41] and GROMACS v.2024.4 [42]. For the force fields, FF19SB was applied to proteins, TIP3P to water molecules, and GAFF2 to itraconazole. Regarding heme, a cofactor located in the active center of CYP51, quantum mechanical calculations were performed by Gaussian v.16 (Gaussian, Inc., Wallingford CT, USA) using the density functional theory (DFT) at the B3LYP/6-31G* level [43], and the resultant model were subsequently integrated into the MD simulations. The system was equilibrated under in the NVT ensemble for 1 ns, followed by NPT equilibration to stabilize the pressure at 1 bar and temperature at 300 K. Finally, the MD simulations were run for 150 ns, during which the temperature was maintained at 300 K using the V-rescale thermostat, and the pressure at 1 bar using the Parrinello−Rahman barostat. Particle Mesh Ewald (PME) was used to calculate electrostatic interactions, with cutoff distances of 10 Å for Coulombic, electrostatic, and van der Waals interactions. For the visualization and analysis of data, PyMOL v.3.0.0 (http://www.pymol.org/pymol) was used. The total binding energy between CYP51 and itraconazole were calculated using gmx_MMPBSA v.1.6.2 [44].

**2nd Manuscript: Pages 19-20, Lines 396-406**

The protein models of MFCYP51A1 and MFCYP51A2 were successfully constructed using AlphaFold2 and refined with OpenMM. The results indicated that the active sites of both enzymes are well-conserved, as shown in Fig. 6. Docking simulations demonstrated that itraconazole binds to the active sites of both MFCYP51A1 and MFCYP51A2, as shown in Fig. 7. However, the orientation of the nitrogen atom in itraconazole relative to the heme iron differs between the two proteins, as shown in Fig. 7. Furthermore, MD simulations demonstrated that the average distance between the heme iron and the nitrogen atom in itraconazole was 2.67 Å for MFCYP51A1 and 3.47 Å for MFCYP51A2, as shown in Fig. S6. The total binding energies for itraconazole of MFCYP51A1 and MFCYP51A2 were -71.38 and -71.69 kcal/mol, respectively.

**2nd Manuscript: Pages 19-20, Lines 432-440**

Regarding protein modeling, the result of MD simulation demonstrated that the distance between heme iron and the nitrogen atom of itraconazole in MFCYP51A2 was greater than MFCYP51A1, while the total binding energies of the two enzymes are nearly equal (Fig. 7). Previous studies reported that the iron-nitrogen distance is an important factor to form the Fe-N coordination bond essential for azole inhibition [51,52]. Thus, our result suggests that MFCYP51A2 exhibits a weak interaction with itraconazole in comparison with MFCYP51A1, which is consistent with the differences in itraconazole susceptibility between *S. cerevisiae* transformants expressing *cyp51A* genes.

**(Q3)**

Since crystal structures are available for fungal CYP51, it could be useful and help to improve the impact of the manuscript, to construct AlphaFold homology models for Mfcyp51A1 and A2 for docking of the azole inhibitors and comparison of the two structures. This would significantly help to round out the manuscript.

**(A3)**

Thank you for your suggestion. Please read **(A2)**, which also gives the rigid data to answer this question.

**(Q4)**

Figure 1 is quite blurry, at least in my copy of the manuscript. If this figure is to be included in the final manuscript, please be sure that it has significant resolution to be legible.

**(A4)**

Thank you for your careful check of the manuscript. We revise the figure and uploaded it.

**(Q5)**

In the Materials and Methods, the authors state: “The E. coli strain was cultured in LB medium with the addition of 100 mg/L of ampicillin when necessary” please define the term “when necessary” as this is inherently ambiguous

**(A5)**

The corresponding sentences were revised as follows:

**2nd Manuscript: Page 5, Lines 110-111**

The *E. coli* strain was cultured in LB medium with the addition of 100 mg/L of ampicillin to maintain a plasmid.

**(Q6)**

Please check reference format for reference number 39. I could not find the name of the journal in this citation.

**(A6)**

We revised the reference format for the corresponding paper, as Ref. No. . Please check it

**(Q7)**

At four figures, the manuscript is a bit data light for a research manuscript; more indicative of a communication (see point #3 above).

**(A7)**

By revisions, we added the data and results into 2nd manuscript. We believe that they fulfill your suggestions and greatly improve the paper. Thank you for your valuable comments.

**Other Reactions**

According to the other reviewer’s comments, we revised the manuscript as described as follows:

**(R1)**

We newly added the data and descriptions to evaluate the quality of genome assembly (Supplementary Figs. S1 and S3), as follows:

**2nd Manuscript: Page 14, Lines 311-317**

Five of the scaffolds contained telomeric sequences at both ends, while the remaining scaffold a telomeric sequence at one end, as shown in Fig. S1. Genome annotation identified 11,334 protein-coding genes, 180 tRNAs, and 79 rRNAs. The BUSCO v.5.5.0 [45] score based on the eukaryote and sordariomycetes databases were 100% and 99.6%, respectively. These results indicated that a high-quality genome assembly of *M. fahalii* IFM 68171 was obtained

**2nd Manuscript: Page 15, Lines 330-332**

Notably, no homologous *Mfcyp51A2* gene exists in the genome of *M. mycetomatis*, although its adjacent gene locus is conserved, as depicted in Fig. S3.

**(R2)**

We newly designed qPCR primer targeting tubulin gene as a reference and preformed qPCR assay. Then, *cyp51A* genes are quantified using two reference genes (actin and tubulin), as shown in Table 2. In addition, the primers’ efficiency was determined by plotting the standard curves. The results and descriptions are added as follows:

**2nd Manuscript: Page 10, Lines 220-222**

The transcription levels of *cyp51A* were measured using primer pairs Pr3-Pr4 or Pr5-Pr6, and they were normalized against actin and tubulin, amplified with primer pairs Pr7-Pr8 and Pr9-Pr10, respectively.

**2nd Manuscript: Page 17, Lines 358-367**

The transcriptional activity of the *Mfcyp51A* genes in *M. fahalii* was analyzed both in the presence and absence of 2 mg/L itraconazole. To estimate the transcriptional levels, two housekeeping genes (actin and tubulin) [13] were used for internal standards. The primer efficiency was determined using cDNA solutions by plotting a standard curve that were serially diluted by 10-fold, as shown in Fig. S4 and Table S3. The results of qPCR was summarized in Table 2. In the absence of itraconazole, the transcriptional level of *Mfcyp51A1* was 1.89 times higher than that of *Mfcyp51A2*. However, upon the addition of itraconazole, transcription levels of *Mfcyp51A1* increased by 1.25- or 1.96-fold while those of *Mfcyp51A2* increased by 2.00- or 3.14-fold.

**(R3)**

A new author (Dr. Wei CAO) was added into the authorship due to his total contribution to protein modelling and calculations.

**(R4)**

The Table 1 in previous version manuscript, which lists the primer sequences, was transferred into Supplementary Table (Table S1).
